# From Spheroids to Tumor-on-a-Chip for Cancer Modeling and Therapeutic Testing

**DOI:** 10.3390/mi16121343

**Published:** 2025-11-27

**Authors:** Maria Veronica Lipreri, Marilina Tamara Totaro, Nicola Baldini, Sofia Avnet

**Affiliations:** 1 Biomedical Science, Technologies, and Nanobiotechnology Lab, IRCCS Istituto Ortopedico Rizzoli, 40136 Bologna, Italy; mariaveronica.lipreri@ior.it; 2 Department of Biomedical and Neuromotor Sciences, University of Bologna, 40138 Bologna, Italy; marilina.totaro2@unibo.it (M.T.T.); nicola.baldini5@unibo.it (N.B.)

**Keywords:** spheroids, microfluidics, drug screening, sarcomas, carcinomas

## Abstract

The high failure rate of anticancer drugs in clinical trials highlights the need for preclinical models that accurately reproduce the structural, biochemical, and mechanical complexity of human tumors. Conventional two-dimensional cultures and animal models often lack the physiological complexity required to predict clinical outcomes, driving the development of three-dimensional systems that better emulate the tumor microenvironment. Among these, microfluidic-based spheroid models have emerged as powerful tools for cancer research and drug screening. By integrating 3D spheroids with microfluidics, these platforms allow precise control of nutrient flow, oxygen gradients, shear stress, and interstitial pressure, while supporting co-culture with stromal, immune, and endothelial cells. Such systems enable the investigation of drug response, angiogenesis, metastasis, and immune interactions under dynamic and physiologically relevant conditions. This review summarizes recent advances in microfluidic spheroid models for cancer, covering both carcinomas and sarcomas, with an emphasis on device design, biomaterial integration, and translational validation. Key challenges remain, including technical complexity, scalability constraints, and the absence of standardized protocols. Overall, the merger of microfluidic technology with 3D spheroid culture provides a promising pathway toward predictive, ethical, and personalized preclinical testing, bridging the gap between in vitro modeling and clinical oncology.

## 1. Introduction

Cancer remains one of the leading causes of death worldwide, claiming millions of lives each year. This high incidence and mortality emphasize the urgent need for more effective therapeutic strategies. Currently, fewer than 4% of anticancer drug candidates successfully advance through clinical trials to receive FDA approval, largely due to limited efficacy and adverse side effects [1]. A major contributor to this high failure rate is the lack of preclinical models that accurately replicate the complexity and heterogeneity of human tumors. Therefore, developing more reliable and predictive models is essential to accelerate drug development and deepen the understanding of cancer biology [1].

For decades, conventional two-dimensional (2D) cultures and animal models have been the standard tools for preclinical cancer research [2]. However, 2D in vitro systems suffer major limitations: failure to reproduce the extracellular matrix (ECM), realistic cell–cell or cell–ECM interactions, and key biochemical and biomechanical cues critical for recapitulating the tumor microenvironment (TME). Consequently, these models poorly reflect in vivo tumor behavior [3].

Animal models, while offering greater biological complexity, pose significant challenges. They are costly, time-consuming, and difficult to manipulate to precisely tune the TME. Moreover, their translational relevance is limited by interspecies differences in genetics, protein regulation, and immune functions. Ethical concerns further restrict their use [2]. In addition, the compromised immune systems of mice used in xenograft models can alter cancer progression and reduce the reliability of drug testing [2].

Over the past decades, preclinical cancer modeling has evolved substantially, and these limitations have prompted a shift toward advanced three-dimensional (3D) in vitro systems that faithfully reproduce tumor biology, including spheroids, organoids, 3D bioprinted cancer-like tissues, and tumor-on-a-chip technologies. While 2D cultures remain useful for basic mechanistic studies and high-throughput screening, 3D in vitro models better replicate tumor architecture, composition, and microenvironment, providing improved physiological relevance and predictive power [4]. It has been found that 3D models bridge the gap between oversimplified 2D cultures and complex animal studies, offering an ethical, practical, and biologically meaningful platform for cancer research and drug testing [5]. They preserve natural cell morphology and polarity, support realistic cell–cell and cell–matrix interactions [6], provide ECM components, and hierarchical cell organization, and reproduce gradients of oxygen, nutrients, biochemical signaling, and mechanical stress that all contribute to tumoral heterogeneity, tumor growth, invasion, and drug resistance, generating data that are more predictive of clinical responses than those obtained from 2D monolayers [6]. Moreover, 3D systems are generally more stable and support longer culture lifespans, making them ideal for long-term studies of tumor progression and therapeutic evaluation.

Among the available 3D cancer models, tumor spheroids remain the most widely used, as their morphological, functional, and compositional features closely recapitulate key aspects of solid tumors, including hypoxic gradients, nutrient diffusion barriers, ECM deposition, and heterogeneous cell populations [1,3,6,7]. Their simplicity, scalability, and compatibility with many analytical techniques have made spheroids a fundamental tool in cancer biology and drug screening [8]. In details, spheroids mimic the tumor architecture by featuring a central necrotic core, a senescent layer, and a proliferative outer layer, thereby creating gradients of oxygen and pH similar to those found in vivo [3]. Spheroids can be composed of multiple cell types, allowing them to recapitulate the cellular microenvironment of tumors, which includes not only cancer cells but also stromal cells such as mesenchymal stem cells (MSCs), fibroblasts, endothelial cells, immune cells, and signaling molecules like growth factors and cytokines. Compared to organoids, which are more complex and better preserve tissue architecture and genetics, spheroids are simpler, faster to produce, and more scalable for routine drug testing. Additionally, unlike scaffold-based and 3D-printed models, spheroids benefit from having their ECM secreted by the cells themselves, rather than being exogenous. Furthermore, spheroids help to avoid ethical issues associated with animal models, are less costly, and enable controlled, high-throughput experiments [3].

In recent years, the integration of tumor spheroids with microfluidic technologies has given rise to a new generation of advanced in vitro tumor models. Incorporating spheroids into micro-engineered enables the production of more homogeneous and reproducible spheroids, while also reproducing the intrinsic characteristics of the tumor mass and emulating the surrounding (TME) with unprecedented spatial and temporal precision. As a result, microfluidic spheroid-based systems have emerged as highly promising tools for preclinical research, particularly for drug screening applications where reliability and predictive value are critical [8].

This review summarizes recent advances in spheroid generation methods, with a particular emphasis on microfluidic strategies, and examines state-of-the-art tumor-on-a-chip systems as reliable platforms for modeling cancer progression and evaluating therapeutic responses. This integrative perspective provides a coherent framework for understanding how microfluidics can not only generate physiologically relevant spheroids but also enhance their biological fidelity by reproducing key TME components. We highlight major innovations, representative designs, and biological applications, while also addressing current limitations and future challenges in this rapidly evolving field. Together, these insights underscore the transformative potential of micro-engineered 3D models in bridging the gap between conventional in vitro assays and in vivo physiology.

## 2. Current Methods to Obtain Cancer Spheroids

As versatile and widely adopted 3D models, cancer spheroids can be generated using a broad range of fabrication techniques, each offering distinct advantages and limitations depending on the biological question and experimental constraints. Over the years, numerous approaches—ranging from simple static methods to more sophisticated engineered systems—have been developed to promote controlled cell aggregation and spheroid maturation. These methods differ not only in their ability to regulate spheroid size, uniformity, and microenvironmental conditions but also in reproducibility, scalability, ease of use, and compatibility with downstream assays.

### 2.1. Traditional Methods to Obtain Cancer Spheroids

Traditional methods such as spinner flasks promote cell aggregation through continuous stirring and are suitable for large-scale, long-term cultures. However, they often produce spheroids of inconsistent size and expose cells to shear stress, which may induce apoptosis [9,10,11]. Simpler approaches like the hanging drop method and ultra-low attachment (ULA) plates are widely used due to their ease of use, reproducibility, and compatibility with a broad range of cell types. Despite their accessibility and low cost, these methods face challenges such as limited scalability, evaporation risk, and inconsistent spheroid size [12,13]. Techniques like the liquid overlay method and magnetic levitation offer improvements in spheroid formation and uniformity, yet they remain sensitive to cell type or require additional steps such as nanoparticle labeling, which may alter cell behavior [9,12,13,14,15]. Gel embedding provides a more physiologically relevant environment by mimicking the ECM, supporting the development of complex, tissue-like structures. However, variability in hydrogel properties and the need for advanced imaging tools can limit its practicality [16,17].

### 2.2. Microfluidic Strategies for Spheroid Generation

To solve the challenges posed by the use of traditional methods, microfluidics has emerged as a cutting-edge technology for spheroid formation. Rather than representing a single technique, microfluidics encompasses a family of strategies that leverage fluid dynamics, microscale geometries, and biomaterial integration to precisely guide cell seeding, confinement, and aggregation. This versatility enables the generation of reproducible, physiologically relevant spheroids under highly controlled chemical and mechanical cues, overcoming many limitations of traditional methods.

The following sections provide an overview of the principal techniques currently employed to generate cancer spheroids, outlining their respective strengths and weaknesses.

Microfluidic platforms enable the generation of spheroid tumor models either in situ—by seeding single cells directly on-chip—or by the integration of pre-formed spheroids produced externally. Although they require specialized equipment and technical expertise, by leveraging fluid dynamics, biomaterial properties, and microstructured surfaces, these systems offer precise control over spheroid size and architecture.

**Figure 1:**
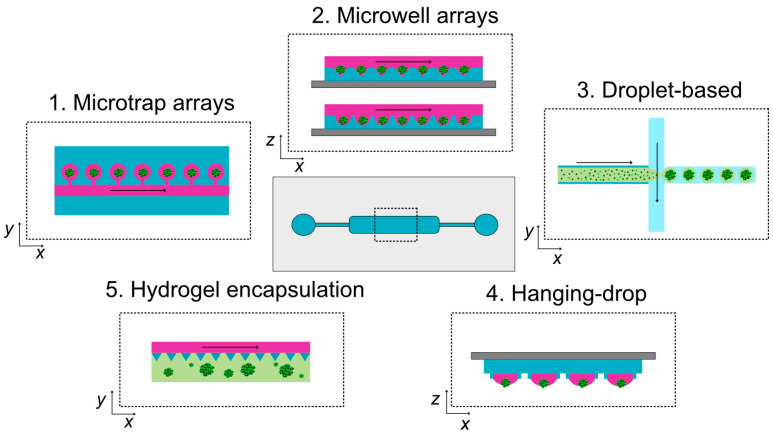
Schematic representation of the main techniques for tumor spheroid generation in microfluidic devices. Central scheme: generic microfluidic device showing inlets, outlets, microchannels, and a central culture chamber, whose geometry varies with the spheroid formation technique. Insets (top *xy* or side *xz* views) illustrate representative approaches: (1) microtrap arrays—cells guided by flow are captured in microchambers and aggregate into spheroids; (2) microwell arrays—cells settle by flow and gravity into U-bottom or cone-shaped wells, forming spheroids; (3) droplet-based microfluidics—cells encapsulated within uniform aqueous or hydrogel droplets via flow-focusing or T-junctions (light green = hydrogel); (4) hanging drop method—cells sediment by gravity and aggregate at the droplet bottom; (5) hydrogel encapsulation—cells embedded within 3D hydrogels proliferate and self-assemble into spheroidal microtissues. Black arrows: perfusion direction.

For spheroid generation, various techniques are available for spheroid formation within microfluidic devices, each offering specific benefits in terms of morphology, microenvironment control, assay compatibility, imaging accessibility, and physiological relevance. The most commonly employed strategies include microtrap and microwell arrays, droplet- and perfusion-based systems, hanging drop techniques, and hydrogel encapsulation (Table 1, Figure 1).

In conclusion, microfluidic platforms offer versatile and precise systems for generating tumor spheroids under controlled and dynamic conditions, enhancing reproducibility and experimental relevance. Despite variations in throughput and complexity, these approaches mark a significant advancement toward more predictive, scalable, and integrative in vitro cancer models.

## 3. Engineering the Tumor Microenvironment with Microfluidic Platforms

**Figure 2:**
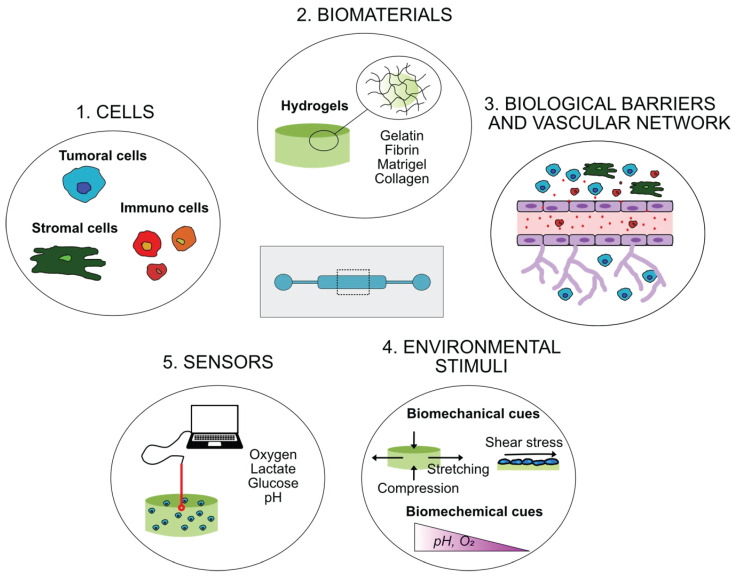
Schematic representation of the main components required to mimic the tumor microenvironment (TME) within a microfluidic device: (1) cells, including tumor cells and key stromal populations such as mesenchymal stem cells, fibroblasts, and endothelial cells (e.g., HUVECs); (2) biomaterials, primarily injectable hydrogels such as gelatin, fibrin, Matrigel, or collagen, which serve as extracellular matrix (ECM) mimics; (3) biological barriers, including blood vessel wall, vascular networks, and specialized interfaces such as the blood–brain barrier; (4) environmental stimuli, both biomechanical (compression, stretch, and shear stress) and biochemical (pH, oxygen gradients, nutrients), that regulate tumor behavior and response to therapy; (5) integrated sensors enabling real-time monitoring of cellular metabolism and microenvironmental cues, with the possibility of implementing feedback loops to dynamically adjust stimulus levels.

For all the techniques described in Section 2.2, with the exclusion of hydrogel encapsulation, microfluidic devices may function only as tools for spheroid fabrication, with the resulting 3D structures subsequently used as stand-alone models. However, in tumor modeling—particularly in approaches involving spheroid generation and culture—microfluidic technology can serve a dual role, i.e., enabling spheroid formation while recreating physiological conditions that mimic tumor progression in vivo. These systems have also been effectively applied to high-throughput drug screening and chemotherapy testing, including with patient-derived tumor cells [22,25,28,34,35]. In such cases, tumor-on-a-chip platforms integrate microfluidics to reproduce dynamic physiological parameters such as nutrient flow, cell–cell interactions, biological barriers, biochemical gradients, and mechanical forces, thereby allowing precise manipulation of the TME. Importantly, these systems can incorporate multiple functional components (Figure 2):

• Multiple Cell Types: Tumor-on-a-chip devices can host various cell populations—tumor, stromal, and immune cells—mimicking both primary and metastatic niches. Stroma-rich or heterotypic spheroids often display reduced drug penetration and increased chemo resistance, underscoring the importance of stroma-derived ECM and stromal components in predictive modeling [28,36,37]. Interestingly, these systems are increasingly used to study tumor–immune interactions and test immunotherapies, including checkpoint inhibitors [38], and enable personalized applications using patient-derived cells thanks to their miniaturization and low cell requirements [22,34,39].
• Biomaterials: Incorporating biomaterials that mimic the native ECM is crucial. Injectable hydrogels such as collagen, Matrigel™, GelMA, and fibrin can be functionalized with ECM components like glycosaminoglycan, elastin, laminine, fibronectin, and various collagens (I, II, IV). Inorganic elements such as hydroxyapatite nanocrystals (in stoichiometric or biomimetic forms) can also be added to reproduce tissue-specific properties, such as bone rigidity [40,41,42]. ECM composition strongly affects stiffness, oxygen gradients, interstitial flow, nutrient diffusion, spheroid morphology, proliferation, and chemosensitivity [43,44].
• Environmental Stimuli: Microfluidic systems allow the application of mechanical (shear, tensile stress) and biochemical (oxygen, pH gradients) stimuli, essential in replicating the dynamic and heterogeneous nature of tumors [37,45,46].
• Vascular Network: These platforms support the formation of vascular-like structures that enhance nutrient and waste exchange, increasing the physiological relevance of the model [47,48]. They also enable detailed studies of angiogenesis and metastasis under controlled flow conditions [26,49].
• Biological Barriers: Devices can replicate key physiological barriers, such as endothelial, epithelial, or blood–brain barriers, allowing investigation of invasion, metastasis, and drug permeability—for instance, in glioblastoma (BBB models) or metastasis research (endothelial permeability) [50].
• Multiple Layers: Multilayer, perfused platforms facilitate high-throughput spheroid generation under hypoxic or oxygen-gradient conditions, supporting studies on reactive oxygen species (ROS) production and drug efficacy in physiologically relevant environments [43,44].
• Sensors: Integrated sensors enable monitoring of chemical and mechanical parameters within the TME—such as oxygen, pH, and shear stress—and real-time detection of cell-derived factors like cytokines, and signaling molecules [51]. Sensor-equipped tumor-on-a-chip systems have also been used for continuous measurement of metabolites, like glucose, lactate, and oxygen levels, providing quantitative insights into metabolism and drug response to agents such as doxorubicin, paclitaxel, fulvestrant, 4-hydroxy-tamoxifen (4-OHT), and tamoxifen [39,52].

Microfluidic systems are also compatible with advanced imaging technologies, enabling high-resolution visualization and real-time monitoring of structural and functional changes in tumor spheroids [22]. For example, Olofsson et al. developed a multi-array microfluidic platform designed for multicellular tumor spheroids that supports automated bright-field image-based analysis of drug-treated spheroids, as well as high-definition deep-tissue confocal imaging of entire MCTS following mounting in a refractive-index-matching medium [53]. This dual-imaging capability allows precise quantification of treatment responses while preserving spheroid integrity, demonstrating how microfluidics can be seamlessly integrated with advanced microscopy for comprehensive spheroid characterization.

In conclusion, microfluidic tumor-on-a-chip systems, combined with tumor spheroid and non-destructive imaging, offer a dynamic and controllable platform for studying, complex interactions within the TME. They support longitudinal assessment under physiologically relevant conditions and enable precise drug delivery—either uniform or gradient-based—facilitating comprehensive dose–response studies. By integrating multiple cell types, ECM-like biomaterials, vascular structures, and physiological stimuli, these models bridge the gap between simplified in vitro assays and complex in vivo environments, substantially enhancing the predictive power of preclinical cancer research.

## 4. State-of-the-Art Tumor Spheroid-on-Chip Platforms for Drug Screening

**Figure 3:**
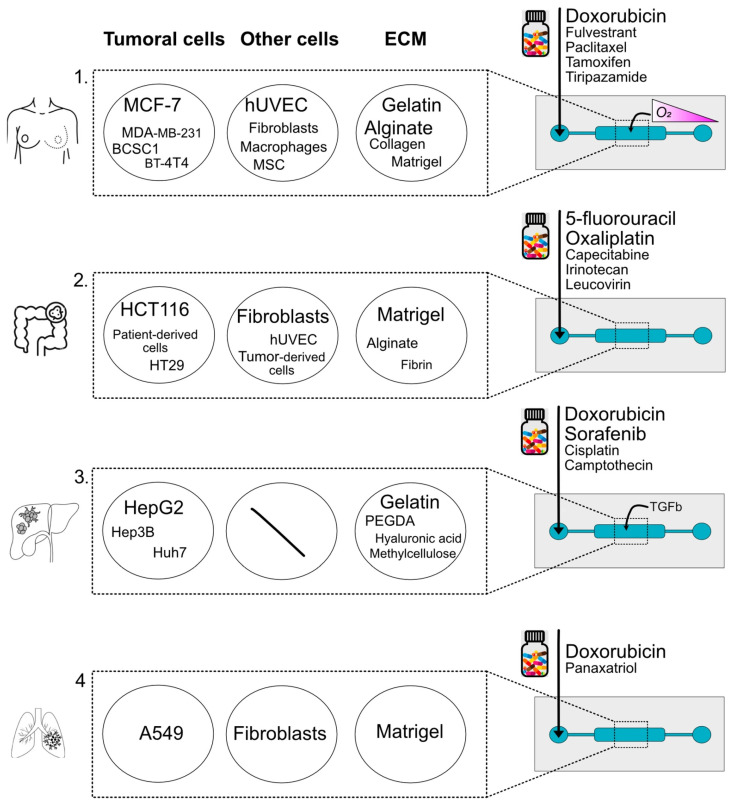
Visual representation of the main components of breast (1), colon (2), hepatic (3), and lung (4) cancer-on-a-chip models and their use of drug screening. For each system, the relevant cell types, ECM components, stimuli, and drugs used are reported. The font size reflects the frequency with which each element has been cited in the literature (PubMed, last 5 years).

Spheroid culture within microfluidic devices has become a leading approach in cancer modeling, offering enhanced physiological relevance and adaptability for multiple tumor types. As described above, these systems enable controlled 3D tumor growth, microenvironmental manipulation, and real-time analysis, making them invaluable for drug screening and mechanistic studies. This section highlights recent advances in microfluidic spheroid models applied to solid tumors, particularly carcinomas (Figure 3) and sarcomas, with emphasis on the translational applicability in therapeutic evaluation.

### 4.1. Carcinomas

Carcinomas represent the most prevalent class of solid cancers and include breast, colorectal, liver, and lung carcinomas. Their high incidence and clinical relevance have driven the rapid evolution of microfluidic and spheroid-based tumor models.

Breast cancer has been at the forefront of microfluidic spheroid model development, offering a robust platform to study this cancer. Commonly used cell lines include MCF7, which produces uniform spheroids, and MDA-MB-231, which aggregates less efficiently [54,55]. Increasing attention has been devoted to patient-derived and cancer stem cells, notably from triple-negative subtypes, to capture intra-tumoral heterogeneity and tailor therapeutic assessment [3]. Co-culture approaches combining tumor cells with fibroblasts, MSCs, and endothelial cells have recapitulated stroma-mediated signaling, while vascularized microchannels have allowed the investigation of endothelial contributions to drug resistance [34]. These models also have allowed mechanistic studies elucidating tamoxifen resistance driven by endothelial cytokines (IL-8, TIMP-1) and TNF-α/NF-κB/mTOR signaling, identifying potential reversal targets, and showing that nanocarrier and polymer–lipid hybrid delivery systems improve spheroid penetration, acidity-specific targeting, and drug uptake, underlining the value of microfluidic models for testing advanced therapeutics [54].

Colorectal cancer (CRC) is a leading gastrointestinal malignancy with pronounced histological and microenvironmental heterogeneity. However, compared to breast cancer, CRC microfluidic spheroid studies are fewer and less standardized. The HCT116 line is most widely used [56], though patient-derived xenograft (PDX) and primary cells are gaining traction [20]. Most CRC platforms focus on drug screening rather than TME reconstruction, typically employing Matrigel™ as a matrix, with the rare inclusion of alginate, fibrin, or fibroblast co-cultures [56]. Testing with standard agents—5-fluorouracil, oxaliplatin, and capecitabine—has been complemented by techniques enhancing drug efficacy, such as electroporation [57], nanoparticle-based drug delivery [58], and microbubble-mediated sonoporation, which improves intracellular uptake and enables ultrasound-triggered localization [59]. In one example, the co-delivery of 3 μM DXR with microbubbles and ultrasound reduced viability to 48 ± 2% compared with 75 ± 5% for DXR alone. Importantly, these models show high predictive fidelity: HCT116 spheroids exposed to in vivo-like oxaliplatin concentration dynamics reproduced xenograft results across growth, proliferation, and apoptosis markers [60]. Similarly, a 32-chamber multiplexed CRC-on-chip platform generated drug–response curves for PDX spheroids, quantitatively correlating in vitro and in vivo outcomes [61].

Hepatocellular carcinoma (HCC), the predominant primary liver malignancy, is distinguished by a complex vascular architecture and microenvironmental dependency governing disease progression and resistance. Commonly used lines include HepG2, Huh7, Hep3B, and human-induced hepatocytes (hiHeps) [62]. ECM replication is central to HCC model design: gelatin and GelMA hydrogels offer tunable stiffness [63,64], while collagen, fibrin, and gelatin–hyaluronic acid composites reproduce native biochemical and mechanical cues. Matrix properties profoundly affect spheroid behavior. In spheroid-based microfluidic devices, increased stiffness enhanced HepG2 spheroid cohesion, whereas Hep3B spheroids remained small and stiffness-independent. Hyaluronic acid reduced hepatic functionality (albumin, CYP3A4) but elevated EpCAM and CD133, implying stemness and tumorigenicity. Sorafenib-based assays revealed that therapeutic response is highly dependent on HCC subtype, matrix stiffness, and hyaluronic acid content. Furthermore, advanced platforms have recreated microvascular networks resembling peritumoral vasculature [64], supporting studies of migration, angiogenesis, and epithelial–mesenchymal transition (EMT) under TGF-β exposure. Hierarchical hydrogel scaffolds, inspired by hepatic lobules, combine micro- and nanoporous architectures to permit multichannel, gradient-based drug testing [65]. Beyond standard therapies (sorafenib, DXR), novel combinations—such as Aloe vera extracts—have been evaluated for their synergistic and resistance-modulating effects [52,66].

Lung cancer is a leading cause of cancer-related mortality worldwide, emphasizing the need for predictive and physiologically relevant preclinical models. Commonly used cell lines, such as A549, are cultured as monocultures or co-cultured with stromal cells (e.g., MSC, fibroblasts, osteoblasts) and endothelial cells to mimic tumor heterogeneity and microenvironment interactions [67,68]. Advanced microfluidic systems replicate the tumor–vascular interface using HUVEC-lined perfused channels, supporting angiogenesis and endothelial–tumor interactions [69]. Patient-derived cells have also been incorporated to enable personalized assay that more accurately mirror patient-specific drug sensitivity [67]. Similarly to other cancer models, collagen, Matrigel^TM^ and fibrin remain the most frequently used ECM substitutes [68,69]. Drug testing applications span conventional chemotherapeutics, such as DXR, cisplatin, paclitaxel, vinorelbine, and etoposide, as well as combination therapies and advanced delivery strategies. Examples include polymer–lipid hybrid nanoparticles (HNPs) for siRNA delivery [70] and stimulus-responsive nano-carriers like the Aprotinin–DXR conjugate [71], both of which enhance tumor penetration, cellular uptake, and selective accumulation in acidic microenvironments while reducing off-target effects [71]. Co-culture with MSCs or exposure to conditioned media has revealed the importance of paracrine signaling in modulating drug sensitivity [72]. An emerging frontier involves the tumor-associated microbiota: microfluidic co-culture of Pseudomonas aeruginosa and human lung tumor spheroids demonstrated biofilm formation Via Psl exopolysaccharide and pyoverdine, promoting tumor viability, ferroptosis resistance, and metastasis [73]. Combined antimicrobial (tobramycin), ferroptosis-inducing (thiostrepton), and chemotherapeutic (DXR) regimens were shown to disrupt this tumor–microbiota interplay, adding a new mechanistic dimension to lung cancer therapeutics. Notably, validation against in vivo xenograft data has confirmed strong concordance in both efficacy and resistance patterns, reinforcing the predictive accuracy of microfluidic lung tumor models for translational research.

In this chapter, across carcinoma types, we examined microfluidic spheroid platforms that have advanced cancer modeling by facilitating controlled, reproducible, and physiologically faithful studies of tumor biology. Core elements have included representative cellular sources, from established lines (e.g., MCF7, HCT116, HepG2, A549) to patient-derived and stem-like cells, engineered extracellular environments, integrating ECM analogs, stromal and immune components, and gradients of oxygen, nutrients, and signaling molecules, rigorous drug-testing pipelines, spanning standard chemotherapies, targeted agents, combinatorial regimens, and innovative nanotherapeutics, and design innovations that support high-throughput screening, personalization, and enhanced scalability. Such platforms increasingly bridge the gap between in vitro and in vivo outcomes, delivering quantitatively predictive insights into therapeutic response, drug resistance, and tumor–stroma dynamics. Table 2 summarizes representative studies from the past five years employing microfluidic spheroid models in carcinoma drug screening, focusing on systems that replicate at least one TME component.

### 4.2. Sarcomas

Sarcomas constitute a highly heterogeneous group of malignancies of mesenchymal origin, encompassing soft tissue sarcomas (STSs) and rarer subtypes such as osteosarcoma, Ewing sarcoma, chondrosarcoma, and alveolar soft-part sarcoma (ASPS). Their rarity, histological diversity, and the lack of reliable preclinical models have long limited therapeutic development. In recent years, microfluidic and spheroid-based models have emerged as promising tools to better reproduce the TME complexity, enabling systematic analysis of drug response, angiogenesis, and resistance mechanisms under physiologically relevant conditions. Despite these advances, research remains less standardized than for carcinomas, and the replication of TME features is still limited.

In this chapter, we summarize all the contributions of the past five years, presenting the current state of the art in microfluidic and spheroid models applied to drug screening on sarcomas.

Osteosarcoma, the most common primary bone malignancy, displays strong metastatic propensity and limited therapeutic progress. Owing to the scarcity of predictive models, various microfluidic have been developed to generate reproducible 3D spheroids for drug testing. A 3D-printed microwell chip enabled high-throughput, size-controlled spheroid formation of MG63 cells with real-time fluorescence analysis, showing fivefold higher DXR resistance compared with 2D cultures [19]. Complementary approaches, such as FD-FLIM imaging, have quantified oxygen consumption within spheroids, providing noninvasive measures of treatment response [83]. Two studies have incorporated microenvironmental factors to increase physiological relevance. Sarkar et al. [18] developed a microtrap-array-based device producing up to 5000 MG63 spheroids reproduced in vivo-like stress responses, including VEGF-A upregulation under hypoxia, suggesting applications in anti-angiogenic therapy testing. Another approach using PDMS co-culture systems integrating MG63 and endothelial cells further elucidated oxygenation-dependent behavior [84]. Emerging patient-derived models extend this translational capability: an agarose-based microfluidic chip generated osteosarcoma and chondrosarcoma spheroids for personalized screening, revealing higher DXR sensitivity in osteosarcoma within 48 h by using a new viability index combining spheroid size and fluorescence intensity [22]. Other strategies integrate heterotypic co-cultures and acidic perfusion, demonstrating IL-6-dependent migration reversible by tocilizumab [85], while alginate/CMC core–shell microcarriers enabled parallel drug-gradient assays, confirming the increased resistance and invasiveness of 3D spheroids [86].

Soft-tissue sarcoma STS encompass diverse mesenchymal tumors with limited responsiveness to systemic therapy. Microfluidic spheroid models have been applied to analyze drug effects, radiotherapy combinations, and TME influences. Using spheroids from STS117 and STS93 lines, a miniaturized platform identified potent synergy between talazoparib and radiotherapy [87]. One study has emphasized the role of microenvironmental conditions, particularly hypoxia, in shaping treatment outcomes. In leiomyosarcoma (SK-LMS-1) and soft tissue sarcoma (STS117) spheroids grown in microfluidic devices, hypoxic cores confirmed by carbonic anhydrase IX (CAIX) staining enhanced response to radiotherapy when combined with the hypoxia-activated agent tirapazamine, which selectively targeted central regions [88]. Finally, beyond drug testing, ECM stiffness has been demonstrated to modulate invasiveness: patient-derived spheroids embedded in GelMA hydrogels invaded more aggressively under low-stiffness conditions, indicating that matrix compliance and hypoxia jointly drive sarcoma progression [89].

Research on Ewing sarcoma, a pediatric bone and soft tissue malignancy, has also benefited from microfluidic modeling to refine regimen scheduling and testing. In A673 spheroids, administration of etoposide followed by cisplatin after 24 h produced markedly stronger cytotoxicity, highlighting the therapeutic impact of treatment sequencing [90]. Label-free machine learning analyses based on spheroid morphology were later applied to predict treatment sensitivity in both A673 and patient-derived xenograft spheroids, offering an efficient high-throughput alternative to conventional viability assays [91].

Chondrosarcoma is notoriously resistant to conventional chemotherapy, making in vitro modeling particularly relevant. Patient-derived spheroids cultured in microfluidic chips have been used to assess DXR efficacy [22]. Quantitative fluorescence-based morphological analyses confirmed their reproducibility and potential for individualized therapeutic evaluation in this refractory tumor.

Finally, alveolar soft part sarcoma (ASPS) is a rare sarcoma subtype distinguished by its highly angiogenic phenotype. A vascularized ASPS-on-a-chip was engineered by co-culturing tumor spheroids with pericytes and endothelial cells, recreating the leaky vasculature typical of the disease [92]. The system enabled mechanistic studies on Rab27a and Sytl2, regulators of tumor-induced vascular remodeling, and facilitated testing of drug penetration across abnormal vessels. Such models provide valuable platforms for investigating angiogenesis and improving therapeutic delivery strategies in vascularized tumors.

Collectively, recent developments in microfluidic and spheroid-based sarcoma models have expanded experimental possibilities to study drug response and tumor–stroma dynamics under controlled and physiologically relevant conditions. Although the field is less mature than carcinoma modeling, advances in patient-derived samples, microenvironmental simulation, and imaging-based analysis now enable more predictive, mechanistic, and personalized preclinical studies. Continued efforts toward standardization and scalability will be critical to unlock these models’ full potential for translational oncology.

## 5. Current Limitations in Using Spheroids Cultured in Microfluidic Devices

While microfluidic-based spheroid tumor models offer important advantages, they also present several limitations. The main challenges involve technical complexity, limited standardization, restricted manipulation, and material constraints [1,93]. Microfluidic device fabrication requires specialized expertise and equipment such as UV lithography, stereolithography, micromilling, laser cutting, and plasma bonding systems [1,94]. These processes are often time-consuming and difficult to scale up. The most common device material, polydimethylsiloxane (PDMS), is valued for its biocompatibility, transparency, gas permeability, and ease of replication, yet it can absorb small molecules, potentially compromising drug-testing accuracy [1,94]. Consequently, research is shifting toward alternative polymers and thermoplastics, which may require different fabrication methods, such as micromilling. Another critical limitation is the lack of standardized guidelines and regulatory frameworks, which hinders reproducibility and comparability across studies [1,95,96]. This inconsistency not only leads to variable outcomes but also delays the regulatory acceptance of microfluidic spheroid models as validated animal-replacement methodologies in preclinical safety and drug-efficacy testing. As emphasized by the European Commission’s Joint Research Centre (JRC) in its 2025 roadmap for standard organ-on-a-chip (OoC) technologies, international consensus is the key to ensure the reliability, scalability, and regulatory recognition of these innovative biotechnologies [85]. Finally, scalability remains a significant hurdle. The inherent complexity of microfluidic systems and the number of operational variables complicate their use in high-throughput screening and industrial applications [1,95]. To overcome these barriers, advances are focusing on automation, both in device fabrication and model operation, to enable reproducible large-scale applications in drug discovery and toxicity testing. Efforts toward standardization and automation are expected to significantly expand the adoption of these models in research and the pharmaceutical industry.

## 6. Conclusions and Future Perspectives

The integration of tumor spheroids with microfluidic technology represents a major step forward in preclinical cancer modeling and drug screening. These systems outperform traditional 2D cultures by incorporating 3D architecture, stromal and immune components, vascular mimicry, and biomechanical forces such as shear stress. Moreover, advanced devices can simulate biochemical gradients and integrate embedded sensors for real-time monitoring of the TME, bringing in vitro assays closer to in vivo tumor physiology.

Looking ahead, microfluidic spheroid platforms are poised to play a critical role in drug development pipelines, enhancing the predictive value of preclinical studies, reducing costs, and minimizing animal testing. By providing physiologically relevant data, they can improve candidate selection for clinical trials. Their ability to incorporate patient-derived spheroids (PDSs) further supports personalized oncology, enabling individualized therapeutic testing that moves beyond the traditional “one-size-fits-all” approach.

Nevertheless, widespread implementation remains limited by the absence of standardized protocols and universal guidelines. Among carcinomas, breast cancer research has made the most progress toward a consensus on cell types, biomaterials, and microenvironmental parameters, whereas sarcoma models remain less developed due to their rarity and biological complexity. This heterogeneity hampers reproducibility and large-scale validation.

From a regulatory perspective, agencies such as the European Medicines Agency (EMA) are collaborating with developers of New Approach Methodologies (NAMs), including organ-on-a-chip systems, to promote scientific validation, standardization, and integration into regulatory decision-making. These efforts align with the principle of the 3Rs (Replacement, Reduction, Refinement) and the EU Biotechnology Act, supporting a gradual transition away from animal-based testing.

Future priorities include the harmonization of fabrication and operational standards, enhanced interlaboratory reproducibility, and robust regulatory pathways for validation and qualification. Through collaboration between academia, industry, and regulators—as signaled by the JRC roadmap (2025)—microfluidic spheroid technologies are expected to achieve the scalability, credibility, and standardization required to achieve their full potential in preclinical research and precision medicine.

## Figures and Tables

**Table 1:** Techniques for tumor spheroid generation in microfluidic devices: description, advantages, and limitations.

Technique	Description	Pro	Cons	Ref.
**Microtrap Arrays**	Microfabricated wells or traps in a chip capture and aggregate cells into spheroids. Allows parallel formation, size control, and easy imaging	Simple, parallel, easy imaging	Limited to certain cell types; less dynamic environment	[18,19,20,21]
**Agarose Microwell arrays**	Microfabricated agarose wells in a chip capture and aggregate cells into spheroids. Allows parallel formation, size control, and easy imaging	Simple, parallel, easy imaging	Limited to certain cell types; less dynamic environment	[22]
**Droplet-Based Microfluidics**	Cells are encapsulated in uniform aqueous droplets (often with hydrogels) using flow-focusing or T-junctions. Enables high throughput, size control, and co-culture. Can use all-aqueous systems to avoid cytotoxic solvents	High-throughput, uniform size, automation-friendly	May require oil phases or surfactants, device complexity	[23,24,25]
**Hanging Drop Microfluidics**	Microfluidic chips create hanging drops where cells aggregate by gravity, enabling uniform spheroid formation and co-culture setups	Uniform spheroids; co-culture possible	Lower throughput, evaporation risk	[26,27]
**Hydrogel Encapsulation**	Cells are encapsulated in hydrogels (e.g., alginate, Matrigel^TM ^) within microfluidic channels or droplets, supporting 3D growth and mimicking the ECM	Mimics ECM; supports complex structures	Hydrogel handling, potential diffusion barriers	[28,29,30,31,32,33]

**Table 2:** Recent microfluidic 3D models of carcinoma spheroids (breast, lung, colorectal, and hepatocellular) incorporating TME components.

Tumor	Cells	TME	Technique	Drug	Refs.
**Breast cancer**	MCF7	- *Stromal cells*: hUVEC - Vascular endothelial barrier	Microtrap arrays	tamoxifen	[54]
	MCF7	- *ECM*: gelatin methacryloyl hydrogels - *Stromal cells*: fibroblasts	Gel-embedded	DXR	[74]
	BT-474	- *ECM*: RGD-alginate and fibrin - *Stromal cells*: hUVEC - Vascular endothelial barrier	Droplet-based bioprinting	DXR	[75]
	MCF7	- *ECM*: gelatin	Gel-embedded/microtrap arrays	DXR-loaded liposomes	[76]
	4T1 mouse breast cancer cells	- *Stromal cells*: 3T3 fibroblasts	Microtrap arrays	paclitaxel-loaded polymeric micelles	[77]
	MCF7	- *ECM*: dextran-alginate droplets surrounded by polyethylene glycol	Droplet-based microfluidics	DXR	[23]
	MCF7	- *ECM*: thiol–acrylate hydrogel	Gel-embedded/microtrap arrays	fulvestrant	[78]
	MCF7 or MDA-MB-231	- *ECM*: biosynthetic hybrid hydrogels composed of poly(ethylene glycol diacrylate) (PEGDA) covalently conjugated to natural protein (fibrinogen)	Droplet-based microfluidics	DXR	[55]
	Patient breast cancer cells	- *ECM*: collagen	Microtrap arrays	DXR, paclitaxel	[3]
	MDA-MB-231 and MCF-7	- *Stromal cells*: mouse embryonic stem cell line ES-D3	Hanging drop microfluidics	anti-angiogenesis drug	[26]
	MCF7	- *ECM:* collagen and Matrigel - *Stromal cells*: hUVEC	Gel-embedded	paclitaxel	[79]
	BCSC1 eGFP	- *ECM*: Matrigel - *Stromal cells*: MSC - *Real time monitoring*: hypoxia	Gel-embedded	DXR, antimycin A	[43]
	MCF7/patient-derived cells	- *ECM*: gelatin and cellulose nanocrystals	Gel-embedded/microtrap arrays	DXR, 4-hydroxy-tamoxifen	[34]
	MCF7	- *ECM*: alginate - *Immune system*: human M2-polarized macrophages - *Biochemical cue*: hypoxia - *Real time monitoring*: hypoxia	Gel-embedded/microtrap arrays	DXR, tirapazamine	[44]
**Colon cancer**	Patient-derived cells	- *ECM:* Matrigel - *Stromal cells*: cells from tumoral tissue	Microtrap arrays	oxaliplatin, capecitabine	[20]
	HCT116	- *ECM:* alginate - *Stromal cells*: NIH3T3 fibroblasts	Droplet-based microfluidics	5-fluorouracil	[56]
	PDX-derived	- *ECM*: Matrigel	Microtrap arrays	5-fluorouracil, oxaliplatin, irinotecan	[61]
	HCT116, HT29	- *ECM*: Matrigel	Gel-embedded	5-fluorouracil loaded Fe3O4-nanoparticles	[80]
	HCT116	- *ECM*: fibrin - *Stromal cells*: fibroblasts - Vascular network	Gel-embedded	5-fluorouracil, leucovorin and oxaliplatin	[81]
**Hepatic cancer**	Huh7	- *ECM*: agarose gel, methylcellulose	Liquid overlay technique	DXR, sorafenib, cisplatin	[82]
	HepG2, Hep3B	- *ECM*: gelatin, hyaluronic acid - Stimuli: TGF-β	Droplet-based microfluidics	sorafenib	[30]
	HepG2	- *ECM*: polyethylene glycol diacrylate (PEGDA) and methacrylate gelatin (GelMA)	Scaffold-embedded	DXR, camptothecin	[65]
**Lung cancer**	A549	- *Other cells*: primary human osteoblasts or bone metastasis secondary to lung	Microtrap arrays	DXR	[67]
	A549	- *ECM*: Matrigel - *Stromal cells*: MRC-5	Microtrap arrays	panaxatriol	[68]
	A549	- *Bacteria*: Pseudomonas aeruginosa	Microtrap arrays	DXR + antimicrobial tobramycin	[73]

## Data Availability

No new data were created or analyzed in this study.
